# Supplementary material for: Efficient removal of pharmaceuticals from water using graphene nanoplatelets as adsorbent
Source: R Soc Open Sci. 2021 Jan 6;8(1):201076. doi: 10.1098/rsos.201076 (PMC7890490; doi:10.1098/rsos.201076)
Supplement: Table S1;Figure S2 ;Figure S3 [file rsos201076supp1.docx]

**Electronic Supplementary Material Information of Efficient Removal of Pharmaceuticals from Water Using Graphene Nanoplatelets as Adsorbent**

Fatin Ahza Rosli,^1^ Khairulazhar Jumbri,^2^ Abdul Halim Abdullah,^1,3^ Haslina Ahmad,^1,4*^ Sazlinda Kamaruzaman,^1^ and Nor Ain Fathihah Abdullah^2^

^1^Department of Chemistry, Faculty of Science, Universiti Putra Malaysia, 43400 Serdang, Selangor, Malaysia.

^2^Department of Fundamental and Applied Sciences, Faculty of Science and Information Technology, Universiti Teknologi PETRONAS, 32610 Seri Iskandar, Perak, Malaysia

^3^Institute of Advanced Technology, Universiti Putra Malaysia, 43400 Serdang, Selangor, Malaysia.

^4^Integrated Chemical Biophysics Research Centre, Faculty Science, Universiti Putra Malaysia, 43400 Serdang, Selangor, Malaysia.

*Corresponding author: [haslina_ahmad@upm.edu.my](mailto:haslina_ahmad@upm.edu.my)

**Table S1.** Intraparticle diffusion of sulfamethoxazole and acetaminophen onto GNP C300.

|  | **Sulfamethoxazole** | **Acetaminophen** |
| --- | --- | --- |
| **K_id1_** | 0.0718 | 0.0732 |
| **C** | 0.0134 | 0.014 |
| **R²** | 0.8666 | 0.8594 |
| **K_id2_** | 3 x 10^-4^ | 8 x 10^-4^ |
| **C** | 0.1517 | 0.154 |
| **R²** | 0.9251 | 0.9049 |
| **K_id3_** | 5 x 10^-5^ | 2 x 10^-4^ |
| **C** | 0.1505 | 0.1514 |
| **R²** | 0.5657 | 0.7887 |

| **TOP VIEW** | **SIDE VIEW** |
| --- | --- |
| 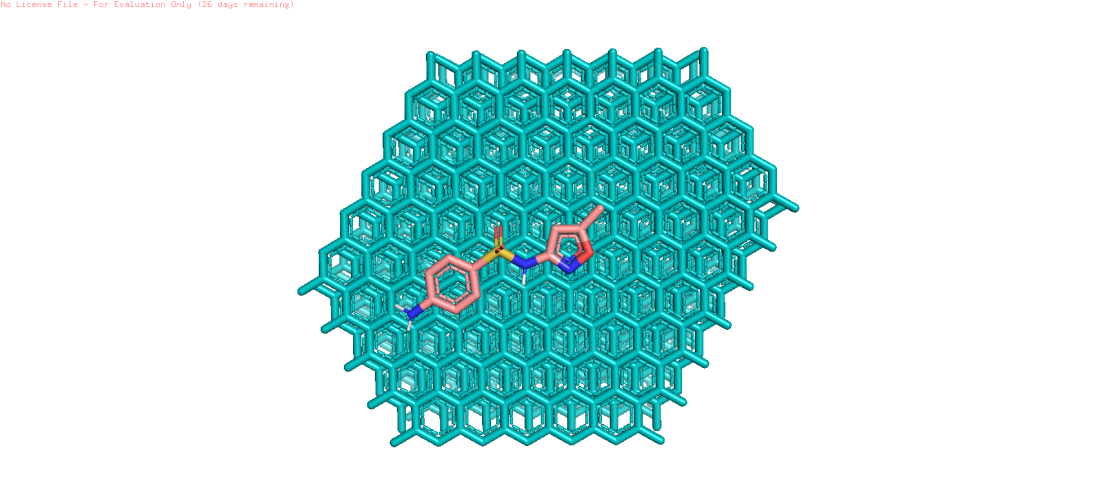 | 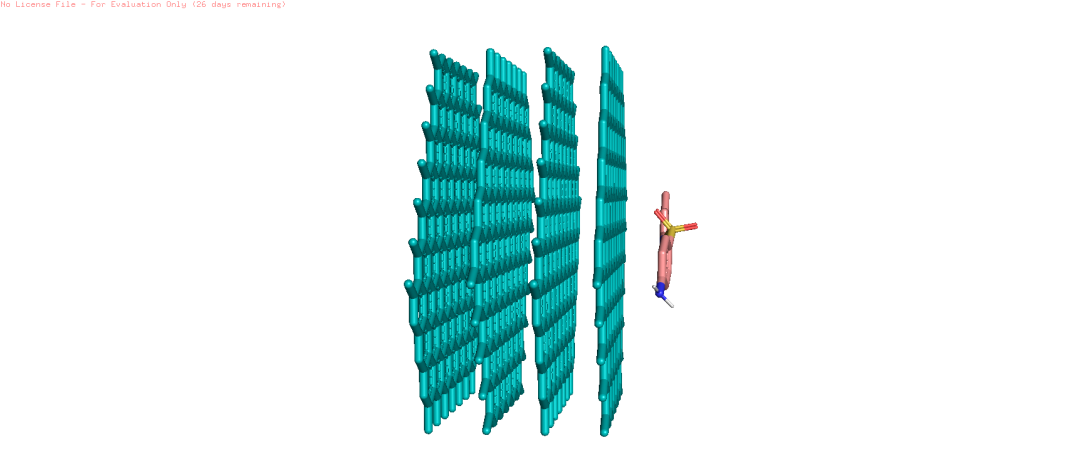 |
| GNP-SMX | |
| **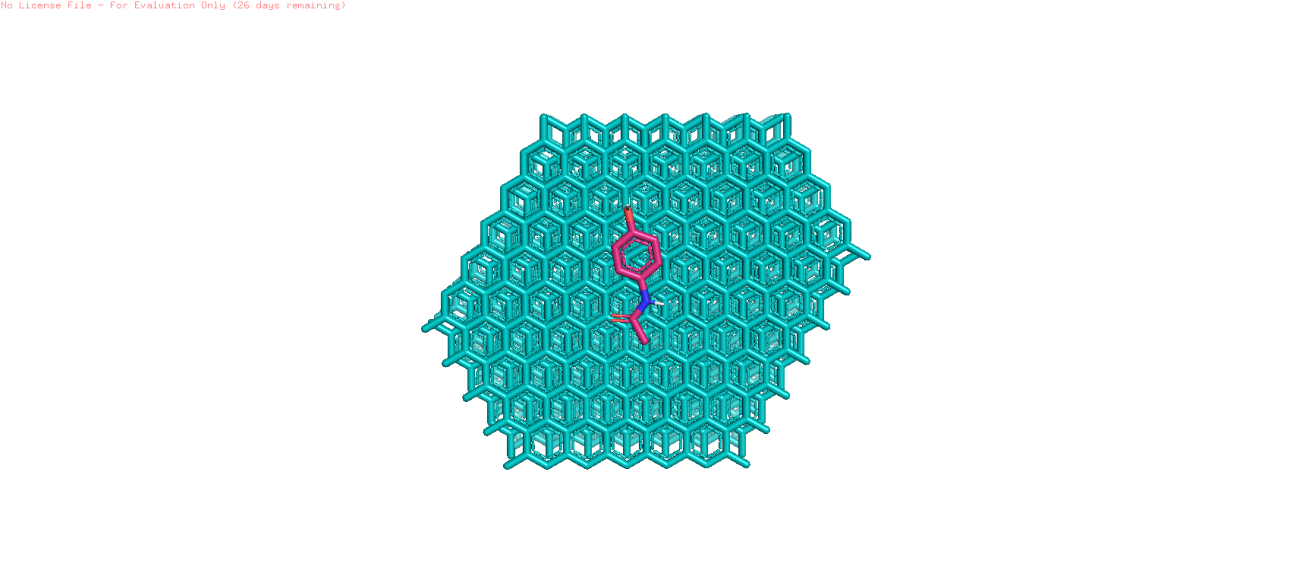** | **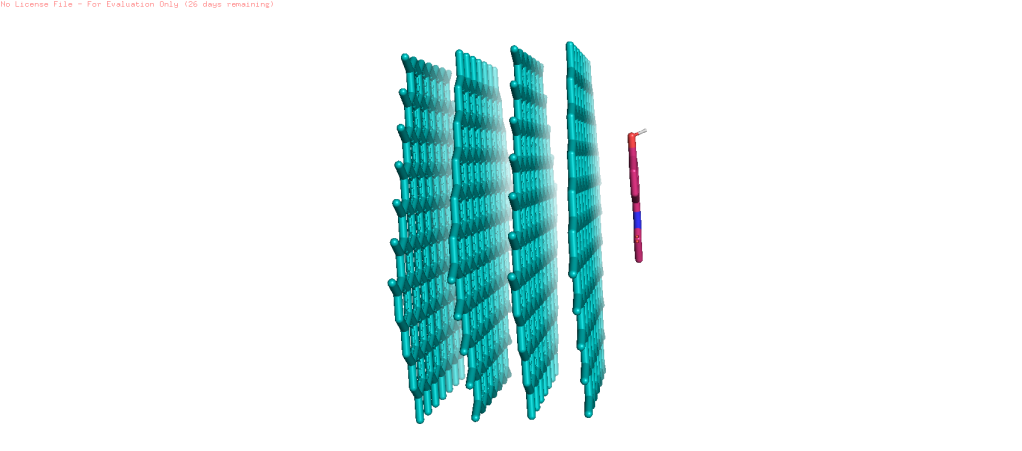** |
| GNP-ACM | |

**Figure S2.** Molecular docking of GNP-SMX and GNP-ACM simulation for adsorption of SMX and ACM on the surface of GNP.


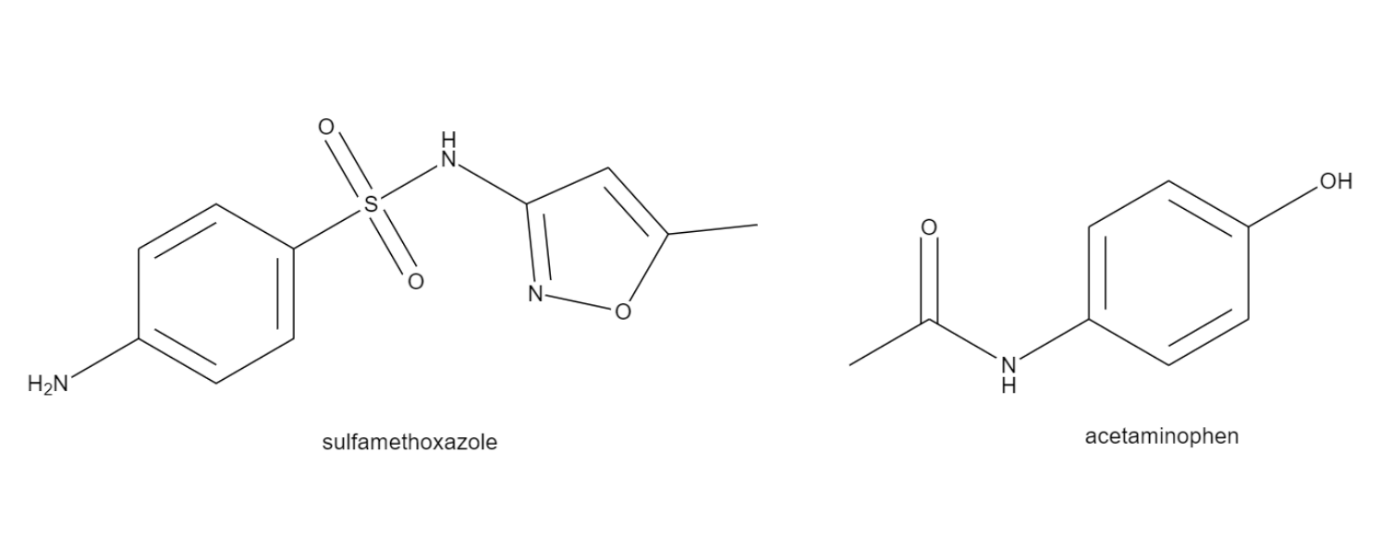


**Figure S3.** Chemical structure of pharmaceuticals used as target pollutants in this research.
